# The effects of certification of head and neck cancer centers on the survival of patients with a head and neck cancer

**DOI:** 10.1186/s12885-026-15624-z

**Published:** 2026-01-26

**Authors:** Olaf Schoffer, Max Kemper, Michael Gerken, Veronika Bierbaum, Christoph Bobeth, Martin Rößler, Patrik Dröge, Thomas Ruhnke, Christian Günster, Kees Kleihues-van Tol, Chia-Jung Busch, Monika Klinkhammer-Schalke, Jochen Schmitt

**Affiliations:** 1 Center for Evidence-Based Healthcare, Faculty of Medicine and University Hospital Carl Gustav Carus, TUD Dresden University of Technology, Fetscherstraße 74, Dresden, 01307 Germany; 2 Department of Otorhinolaryngology, Head and Neck Surgery, Faculty of Medicine and University Hospital Carl Gustav Carus, TUD Dresden University of Technology, Fetscherstraße 74, Dresden, 01307 Germany; 3 Tumor Center - Center for Quality Assurance and Health Services Research, University of Regensburg, Am BioPark 9, Regensburg, 93053 Germany; 4 AOK Research Institute (WIdO), Rosenthaler Str. 31, Berlin, 10178 Germany; 5 Association of German Tumor Centers (ADT), Kuno-Fischer-Straße 8, Berlin, 14057 Germany; 6 Department of Otorhinolaryngology, Head and Neck Surgery, University Medicine Greifswald, Ferdinand-Sauerbruch-Strasse, Greifswald, 17475 Germany

**Keywords:** Head and neck cancer, Certification, Outcome quality, Healthcare-related data, Healthcare research

## Abstract

**Introduction:**

Head and neck cancers represent a heterogeneous group of tumor in terms of both, their entity and location. This includes cancers of the lip, oral cavitiy, pharynx (epi-, oro- and hypopharynx), larynx, nasal cavity/ sinus and salivary gland. Additionally, they are associated with functional impairments that place highly specific demands on treating practitioners. Since the introduction of the German National Cancer Plan in 2008, cancer treatment has been aligned with the latest scientific findings through evidence-based guidelines. Meanwhile, treatment courses and outcomes have been systematically recorded by expanding the German cancer registry. In addition, the certification of treatment centers aims to ensure consistently high-quality care. Given the considerable investment of time, personnel, and financial resources required for certification, the question arises whether patients with head and neck cancer benefit from receiving (initial) treatment at a certified center compared to a non-certified facility.

**Methods:**

We used patient-specific information from statutory health insurance (SHI) and clinical cancer registry (CCR) data for the period 2009–2017 as well as hospital characteristics from standardized quality reports. We applied multivariable Cox regressions to analyze differences in survival between patients treated in hospitals with and without German Cancer Society (DKG) certification.

**Results:**

The sample comprised 52,749 (SHI) and 15,287 (CCR) patients with head and neck cancer who were treated in 872 hospitals (44 DKG-certified, 828 not DKG-certified). 15.5% (SHI) and 25.9% (CCR) of patients were (initially) treated in DKG-certified cancer centers. Cox regression analysis for overall survival showed that patients treated in DKG-certified cancer centers were at significantly lower risk of death (SHI data: HR = 0.94, 95% CI = 0.89–1.00, *p =* 0.043; CCR data: HR = 0.89, 95% CI = 0.83–0.95, *p <* 0.001). Notably, the difference was even more pronounced for recurrence-free survival (CCR data: HR = 0.81, 95% CI = 0.72–0.92, *p =* 0.001).

**Conclusion:**

Although achieving certification requires a substantial investment of personnel, time, and financial resources by the treating institution, it is associated with a measurable survival advantage for patients. Therefore, treatment of head and neck cancer patients should preferably be provided at certified head and neck cancer centers.

**Supplementary Information:**

The online version contains supplementary material available at 10.1186/s12885-026-15624-z.

## Introduction

Head and neck cancers are highly heterogeneous and can involve the lip, oral cavity, entire throat (epi-, oro-, and hypopharynx), larynx, nasal cavity, paranasal sinus and salivary gland. Cervical carcinoma of unknown primary (CUP syndrome) is also included in this group. Depending on their location, these cancers produce a wide variety of symptoms and treatment-related side effects that may require further therapy.

With an incidence of approximately 21.8 per 100,000 people in Europe, the mortality is about 15.6 per 100,000 people [1]. In 2020, 168,000 new cases and 73,000 deaths were reported. According to the latest Cancer Registry figures, 18,628 new cases were registered in Germany in 2023, 55.27% of which were treated in a DKG-certified facility [2].

Although 90% of carcinomas are squamous cell carcinomas, five-year survival rate varies considerably by etiology and location (e.g., 25% for hypopharyngeal cancer and 61% for laryngeal cancer), as well as by stage (90% stage I, 75–80% stage II, 45–75% stage III and up to 50% stage IV) [3]. These epidemiological data underscore the substantial burden of head and neck cancer and the urgent need for high-quality treatment, rehabilitation, and follow-up to detect recurrences early. Beyond cure, quality of life and functional outcomes are increasingly prioritized.

To meet these requirements and to ensure a comprehensive evaluation of all available treatment options with the aim of optimizing outcomes, interdisciplinary and interprofessional discussions and consultations are essential both prior to and throughout the course of treatment. All potential benefits and risks must be carefully assessed, with guaranteed access to multidisciplinary expertise across all therapeutic modalities.

In response, certification programmes were developed in Germany to provide every cancer patient with the best possible cross-sectoral and interdisciplinary diagnosis and treatment based on the latest scientific knowledge. The programmes also aim to make this comparable both nationally and internationally, using measurable criteria.

This approach was driven by data demonstrating considerable variation in survival outcomes among European breast cancer patients, as well as by early efforts to establish certified breast cancer centers from 2003 onwards [4]. In 2008, the Federal Ministry of Health, together with the Association of German Tumor Centers (ADT), the German Cancer Society (DKG), and the German Cancer Aid (DKH), launched the National Cancer Plan to further advance these aims [5]. In the following years, further certification programmes for other cancer types were developed by the relevant professional associations [6].

Several structural initiatives have been implemented to address unmet needs and achieve the outlined goals for head and neck cancer patients. These include certification of head and neck cancer centers to ensure high-quality care, the establishment of cancer registries for systematic case tracking and disease surveillance, and the development of an oncology guidelines programme to generate evidence-based treatment recommendations supported by clearly defined quality indicators. Accordingly, S3 guidelines have been developed in Germany in recent years for oral cavity, laryngeal, oro-/hypopharyngeal carcinomas, salivary gland cancer, and nasal/paranasal sinus cancer [7–10]. In addition, supplementary guidelines have been established for tumor treatment, such as supportive therapy and oropharyngeal dysphagia resulting from head and neck tumor disease [11, 12].

To successfully obtain certification from the German Cancer Society, centers must fulfil a comprehensive set of requirements defined in the guidelines and demonstrated through numerous quality indicators, including the achievement of a minimum treatment volume. Since 2008, these requirements have been progressively refined, specified, and expanded, such that both initial certification and ongoing maintenance entail substantial financial, personnel, and time-related commitments for treatment centers [13, 14].

This raises the question of whether, as observed in breast cancer, treatment in certified centers provides meaningful improvements in treatment quality and outcomes for head and neck cancer patients that justifies the required effort. Following the publication of the overall results of the study on 11 entities by Schmitt et al. (2023, [15]), publications appeared on the positive effect of certification on the survival of patients with pancreatic cancer [16], colorectal cancer [17, 18], breast cancer [19], uterine cancer [20], and lung cancer [21] in Germany.

The following manuscript examines the association between DKG certification and treatment outcomes in patients with head and neck cancer treated in certified and non-certified facilities.

## Materials and methods

### WiZen-study

The cohort study on the ‘Effectiveness of care in certified cancer centers’ (WiZen) investigated whether certified cancer centers have advantages over non-certified hospitals in Germany in terms of survival of patients with various cancer types. The project was funded by the Innovation Fund of the Joint Federal Committee (G-BA, 01VSF17020). Patients with head and neck, colorectal, pancreatic, breast, lung, prostate, brain, and gynecological cancers were considered. The results for patients with head and neck cancer are presented below.

### Data basis

On the one hand, the data basis was nationwide, anonymized billing data from the statutory health insurance scheme (SHI) for AOK insures for the study period 2006–2017, provided by the AOK Research Institute (WIdO). For analyses service and master data from the following service areas were merged across sectors: insured person master data (§ 288 Social Code Book V (SGB V)), inpatient and outpatient care (§ 295 SGB V, § 301 SGB V) and outpatient medication (§ 300 SGB V). A diagnosis-free phase in the years 2006–2008 was used to determine incident cancer cases, so that the period 2009–2017 was available for analyses.

On the other hand, pseudonymized data from the Clinical Cancer Registries (CCR) in Brandenburg, Dresden, Erfurt and Regensburg formed the data basis. The pooled data set comprised the initial diagnoses from the period 2006–2017 with personal information and disease-specific data. For better comparability with the analysis results of the SHI data, the evaluation collective was limited to the diagnosis years 2009 to 2017.

In addition to the SHI and CCR data, structural characteristics of the hospitals from the publicly accessible structured quality reports in accordance with Sect. 136 SGB V and data on DKG certification of hospitals, including the periods of certification, were used. In the context of the results presented here, patients with treatment (CCR) or index treatment (SHI) in centers certified by the German Cancer Society (DKG) (organ cancer centers and oncology centers) were considered as an intervention group.

The data was pseudonymized at patient and hospital level and transmitted in encrypted form. The pseudonymization at both levels was carried out by the WIdO and the data-providing CCR and subsequently evaluated at the Center for Evidence-based Healthcare (ZEGV) Dresden and the Regensburg Tumor Center (TZR) at the Center for Quality Assurance and Health Services Research at the University of Regensburg. The WiZen study was approved by the ethics committee of the TU Dresden (EK95022019) and registered at ClinicalTrials.gov (NCT04334239). Data processing and analysis were performed in accordance with the Declaration of Helsinki and the General Data Protection Regulation of the European Union.

### Inclusion and exclusion criteria

Patients with a diagnosis age of at least 18 years and a first diagnosis of head and neck tumor (ICD-10-GM: C00-C14, C30-C32; see [22]) in the years 2009–2017 were included. The selection of ICD-10 codes was determined by clinical expert committees. Patients with identical initial diagnosis and date of death as well as those with missing or implausible information on confounders were excluded. Further exclusions were made in the SHI data for patients without continuous insurance with the AOK, without a main inpatient diagnosis (ICD-10-GM) of the entity under consideration and for index treatment in a clinic within one year before the DKG certificate was issued (change of center status). Index treatment was defined as the first entity-specific inpatient treatment with a main or secondary diagnosis of the respective entity.

### Endpoints

The primary endpoint was overall survival from index treatment (for SHI data) or initial diagnosis (for CCR data). First diagnosis was the earliest date with at least clinical confirmation of a cancer diagnosis, but usually the date of first histological confirmation, excluding diagnoses registered as recurrence (CCR). Survival times of patients without a date of death or with a date of death after 2017 were treated as right-censored as of 31 December 2017. If there was a last known date in the CCR data for patients without a date of death with a notification of being alive up to 2017, this was used for right-censoring.

Recurrence-free survival was considered as a further endpoint in the CCR data analysis. In this case, local, regional and distant metastasis recurrence events were added to the ‘death’ event. In the case of consecutive events, only the first recurrence event was taken into account.

### Intervention

Treatment at a DKG-certified center was defined as the intervention. Patients who received their initial treatment at a certified center at this time formed the intervention group, while the others formed the control group. If documented, the date of resection with the main diagnosis of the respective entity was considered the first treatment; otherwise, it was the date of the first hospitalization. For CCR data, the DKG certification status of the treating hospital at the time of initial diagnosis was used if an institution identifier was available. Otherwise, the case-related variable 'center treatment yes', provided by all registers, was used. For hospital networks and hospitals with several sites, as direct assignment was not possible, all hospitals/sites were assigned the status of a DKG-certified hospital if one of the sites had this status.

### Risk adjustment

For risk adjustment of the estimated center effects, the following variables were included as influencing factors at patient level: age (grouped), sex, year of diagnosis or index treatment, and disease severity (SHI: distant metastases, other oncological diseases and comorbidities; CCR: ICD-10 diagnosis, UICC stage, grading and lymph vessel/vein invasion). The selection of entity-specific comorbidities was carried out according to Elixhauser et al. [23], incorporating clinical expertise. For the SHI data, the number of beds, whether the hospital is a university or teaching hospital, and the hospital's ownership were taken into account from the structured quality reports. Socioeconomic data was not available in the database, so it could not be integrated.

### Statistical analysis

To estimate association of certification with the endpoint while taking into account possible explanatory variables/confounders, overall survival was modelled using multivariable Cox regression. Hazard ratios were reported, along with 95% confidence intervals. Cox models also enable the modelling of possible correlation of patient outcomes within hospitals by including a random effect at hospital level within the SHI data [24]. These models are referred to as Cox models with shared frailty. To compare an aspect of the therapeutic decision-making process between certified centers and other hospitals we examined the proportion of resections performed as index treatments in relation to all index treatments and reported the corresponding Clopper–Pearson confidence intervals and p-values of chi-square test based on SHI data. The proportional hazards assumption was verified by visual inspection of the stratified survival curves, as inferential statistical verification methods would consider irrelevant deviations from proportionality to be significant due to the high number of cases.

## Results

**Fig. 1:**
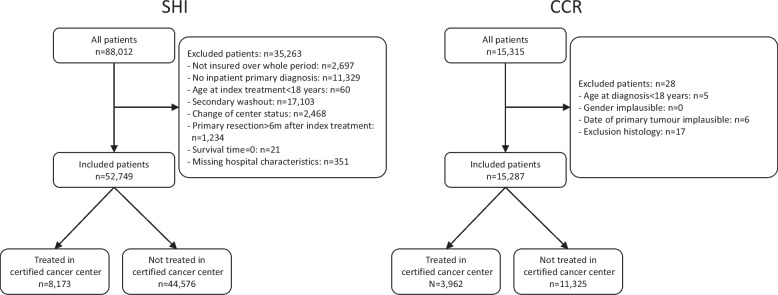
Flow chart showing the patients included and excluded from the relevant data sources (SHI: Statutory Health Insurance; CCR: Clinical Cancer Registry), including the exclusion criteria and the distribution of treatment in certified and non-certified centers

**Fig. 2:**
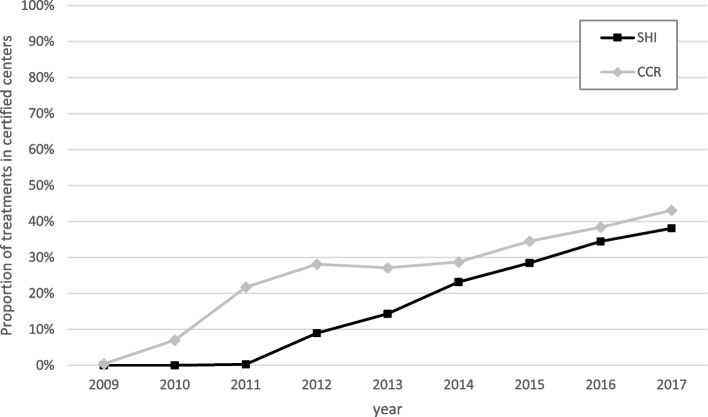
Percentage of patients treated at the center (head and neck cancers – C00-C14, C30-C32) over time according to data source (SHI: Statutory Health Insurance; CCR: Clinical Cancer Registry)

The sample comprised 52,749 patients with head and neck cancer who were treated in 872 hospitals (44 DKG-certified and 828 non-DKG-certified, SHI data), and 15,287 patients with head and neck cancer who were treated in 872 hospitals (44 DKG-certified and 828 non-DKG-certified, CRC data). Of these patients, 15.5% (*n =* 8,173, SHI data) and 25.9% (*n =* 3,962, CCR data) were treated in DKG-certified cancer centers (see Table 1 and Fig. 1). Between 2009 and 2017, the proportion of patients with head and neck cancers treated in DKG-certified centers increased from 0.0% to 38.1% (SHI data) and from 0.0% to 43.1% (CCR data; see Fig. 2). No relevant differences were found between certified and non-certified centers with regard to patient characteristics (age, sex, clinical characteristics). However, the proportion of unknown values in the CCR documentation was lower in certified centers than in non-certified centers, except for grading. In terms of hospital characteristics, larger hospitals were more likely to have certificates than smaller hospitals. Therefore, a possible survival advantage in certified hospitals had to be viewed in light of the different hospital characteristics.

**Table 1:** Analysis populations (head and neck cancers—C00-C14, C30-C32) certified/uncertified, number and percentage by characteristic group for all entities and data sources considered (SHI: Statutory Health Insurance; CCR: Clinical Cancer Registry)

**Characteristic**		**SHI-data**		**CCR-data**	
		**certified**			
	**subgroup**	**yes**	**no**	**yes**	**no**
**Investigation unit: patients**					
	**total (n)**	8,173	44,576	3,962	11,325
Age	**18–59 years (%)**	32.8	35.3	41.7	45.1
	**60–79 years (%)**	54.0	52.9	50.1	47.3
	**80 + years (%)**	13.2	11.8	8.2	7.5
Gender	**female (%)**	26.9	24.9	23.6	21.4
Stage	**UICC I (%)**	-	-	21.2	17.5
	**UICC II (%)**	-	-	11.5	10.1
	**UICC III (%)**	-	-	11.9	12.7
	**UICC IV (%)**	-	-	45.3	40.2
	**unknown (%)**	-	-	10.1	19.4
Localisation	**oral cavity, salivary glands C00-08 (%)**	46.5	41.8	46.8	41.9
	**pharynx C09-14 (%)**	29.0	31.6	32.4	35.2
	**nose, sinuses, ear, larynx C30-32 (%)**	24.5	26.5	20.8	22.9
Distant metastases	**C78/C79 (%)**	10.1	10.1	-	-
Secondary cancer	**C15-26, C33-76, C97 (%)**	39.3	36.0	-	-
Grading	**G1/2 (%)**	-	-	61.1	58.9
	**G3/4 (%)**	-	-	25.2	30.0
	**GX/n.a. (%)**	-	-	13.6	11.1
Lymphatic vessel invasion	**L0 (%)**			38.5	31.9
	**L1 (%)**	-	-	11.4	13.4
	**LX/n.a. (%)**	-	-	50.1	54.6
Vein invasion	**V0 (%)**	-	-	46.8	41.2
	**V1/2 (%)**	-	-	2.7	3.1
	**VX/n.a. (%)**	-	-	50.5	55.7
**Investigation unit: hospitals**					
	**total (n)**	44	828	-	-
Number of beds	**1–299 (%)**	2.3	50.2	-	-
	**300–499 (%)**	11.4	29.8	-	-
	**500–999 (%)**	18.2	17.5	-	-
	**1000 + (%)**	68.2	2.4	-	-

-: Value cannot be determined for the respective data source; n.a.: no information available

**Fig. 3:**
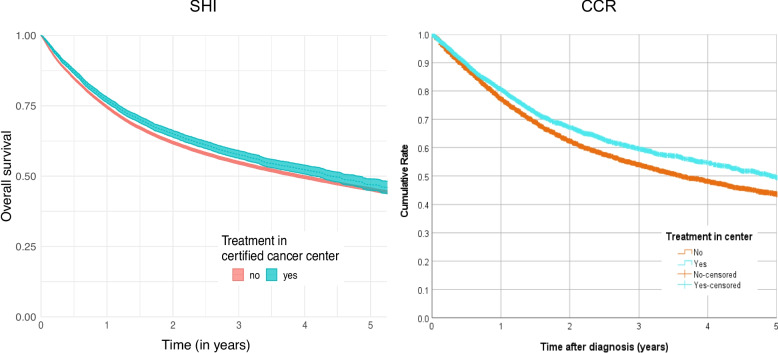
Kaplan–Meier curves showing overall survival of patients (head and neck cancers—C00-C14, C30-C32) by center status (red line non-certified and blue line certified) and data source (left curves: data from SHI (Statutory Health Insurance) and right curve data from CCR (Clinical Cancer Registry)

The unadjusted overall survival rate for patients with head and neck cancer who were treated in certified centers was slightly higher for SHI data and significantly higher for CRC data than for patients who did not receive index treatment in certified centers (see Fig. 3). According to SHI data, the five-year survival rate for treatment in certified centers was 47.0% (95% CI [45.1%–49.1%]) compared to 45.3% (95% CI [44.8%−45.9%]) in non-certified hospitals. The corresponding analysis of CCR data showed 49.5% (95% CI [47.0%–51.9%]) in certified centers vs. 43.6% (95% CI [42.4%–44.8%]) in non-certified hospitals. No visually apparent violations of the proportional hazards assumption were observed with respect to the certification attribute.

The point estimates, including the associated confidence intervals, of the adjusted hazard ratios for certification status on overall survival were below 1 for both SHI and CCR data (Table 2: SHI, HR = 0.94, CI [0.89–1.00], *p =* 0.043; CCR, HR = 0.89, CI [0.83–0.95], *p <* 0.001). Significant survival benefits of 6% (SHI data) and 11% (CCR data) were thus demonstrated for patients in DKG-certified centers for both overall cohorts.

**Table 2:**
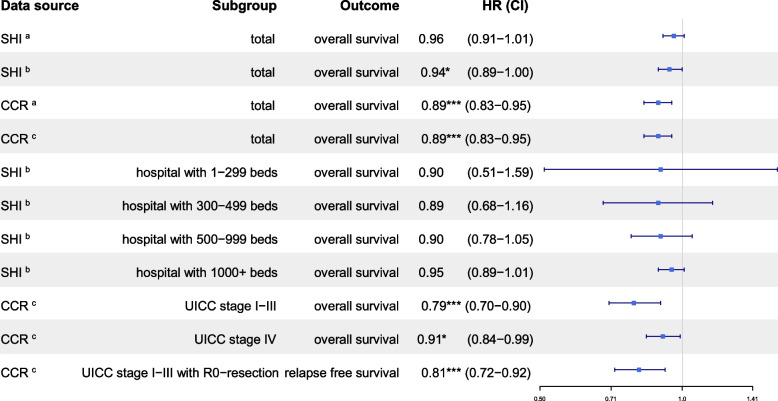
Estimated center effects (head and neck cancers – C00-C14, C30-C32) according to data source (SHI: Statutory Health Insurance; CCR: Clinical Cancer Registry), (sub)group considered and endpoint

*HR* Hazard ratio, CI=95%- confidence interval, *p*-value: *p<0.05, **p<0.01, ***p<0.001

^a^Without adjustment

^b^Adjusted for age, gender, distant metastasis, other oncological diseases, Elixhauser comorbidities, number of beds in the hospital, teaching hospital, university hospital, hospital sponsorship, year of index treatment – dummy coded (based on SHI data)

^c^Adjusted for gender, age at diagnosis, year of diagnosis, ICD-10 diagnosis, stage, grading, lymph node involvement, vein involvement (based on CCR data)

Stratified analyses based on SHI data were conducted to examine possible effect modification by the size of the treating hospital (Table 2). These analyses were stratified by number of beds (1–299, 300–499, 500–999, 1000 +). For all strata, 95% confidence intervals for the estimated hazard ratios of center status overlapped, with all point estimates below 1. Thus, the basic conclusion was unaffected by any possible effect modification due to hospital size. Additionally, the extent to which certification effects were associated with disease severity (stage) and whether results for overall survival can be transferred to recurrence-free survival were investigated for CCR data. The survival benefit of treatment in centers was clearer among patients with localized and locally advanced stages (I–III) than among those with advanced stage IV. A significant survival benefit was observed in both subgroups, with a more pronounced advantage in stages I–III (HR = 0.79; 95% CI = [0.70–0.90], *p <* 0.001) than in stage IV patients (HR = 0.91; 95% CI = [0.84–0.99], *p =* 0.033). Recurrence-free survival was analyzed among patients with R0 resection and no primary distant metastases using the CCR data. The observed benefits were even more pronounced than for overall survival (HR = 0.81; CI [0.72–0.92], *p =* 0.001). Subgroup analyses of the CCR data showed comparable estimates for overall survival for collectives including patients with and without missing information on stage group. Further analysis results for all model specifications can be found in the WiZen final report [22].

The estimation results based on the SHI data were robust when stratified by sex (male/female), presence of other oncological diseases, single hospital/hospital network, presence of distant metastases, tumor resection, and number of hospital beds (< 500/≥ 500) (Supplementary Table 1). The significant survival benefits observed in overall CCR collective were confirmed in most subgroup analyses (Table 2, Supplementary Table 1). Additionally, the survival benefit was more pronounced in DKG-certified centers with a longer certification duration: While the estimated hazard ratio (HR) for cancer centers certified for less than one year was 0.98 (95% confidence interval [CI] = [0.91–1.05], *p =* 0.580), the HR for centers certified for two to less than five years was 0.90 (95% CI = [0.83–0.98], *p =* 0.011) (Supplementary Table 2).

When examining the proportion of resections performed as index treatments, analyses based on SHI data (Table 3) revealed significant differences. Therefore, resection was coded more frequently as an index treatment in non-certified centers (69.9% vs. 73.2%, *p <* 0.001). This also applied to patients up to 79 years of age, but not to older patients. Furthermore, resections were significantly more frequent as an index treatment in non-certified centers for male patients (73.4% vs. 69.5%, *p <* 0.001), for pharyngeal carcinomas (62% vs. 54%, *p <* 0.001), and in hospitals with more than 500 beds (high volume, *p <* 0.001). However, the difference was not significant for smaller hospitals (less than 500 beds, *p =* 0.259).

**Table 3:** Proportion of resection as index treatment certified/not certified, overall and by (sub)group (head and neck cancers – C00-C14, C30-C32, only data from statutory health insurance) and p-values of the corresponding chi-square test

	**Certified**	**Non certified**	***p*-value**
**Proportion of resection as index treatment (all patients)**			
	(*N =* 8,173)	(*N =* 44,576)	
Total	68.6% [67.6%;69.6%]	71.4% [71.0%;71.8%]	< 0.001
**Proportion of resection as index treatment (excluding patients with distant metastases)**			
	(*N =* 7,350)	(*N =* 40,063)	
Total	69.9% [68.8%;70.9%]	73.2% [72.8%;73.6%]	< 0.001
By age group			
18–59 years	74.0% [72.2%;75.7%]	77.8% [77.1%;78.5%]	< 0.001
60–79 years	69.0% [67.6%;70.5%]	72.4% [71.8%;73.0%]	< 0.001
80 + years	63.0% [60.0%;66.1%]	63.1% [61.7%;64.5%]	0.994
By gender			
Female	70.9% [68.9%;72.9%]	72.6% [71.7%;73.5%]	0.122
Male	69.5% [68.3%;70.7%]	73.4% [72.9%;73.9%]	< 0.001
By location			
Oral cavity, salivary glands (C00-08)	73.6% [72.1%;75.1%]	75.0% [74.3%;75.6%]	0.090
Pharynx (C09-14)	54.0% [51.8%;56.1%]	62.0% [61.2%;62.9%]	< 0.001
Nose, sinuses, ear, larynx (C30-32)	80.5% [78.7%;82.3%]	82.8% [82.1%;83.5%]	0.015
According to the number of beds in the hospital			
< 500	74.2% [69.3%;79.1%]	71.1% [70.2%;71.9%]	0.259
> = 500	69.7% [68.6%;70.7%]	74,0% [73.5%;74.5%]	< 0.001

## Discussion

The WiZen study was the first to use such a large cohort to demonstrate the general benefit and entity-specific advantages of treatment in a certified head and neck tumor center compared to a non-certified clinic [15]. Ultimately, the study sheds light on the success of the German National Cancer Plan and the subsequent developments in terms of survival benefit improvement in patient care. Given the lower incidence of head and neck cancers compared to more common malignancies, this study provides — for the first time — large-scale, entity-specific data for this cancer group.

Certification of German hospitals as head and neck tumor centers was initiated relatively late (in 2010/11; [15]). However, the increase in the number of certified centers was more pronounced than for other entities (see Fig. 2). During the observation period up to 2017, the number of certified head and neck tumor centers increased to 44, compared to 827 non-certified clinics [25]. Almost 40% of patients with head and neck malignancies were treated in these centers. Therefore, the observed survival advantage of 6% on SHI data and 11% on CCR data with treatment in certified centers should be interpreted primarily in light of the comparatively late introduction of certification in this entity and the consequently high proportion of recently certified centers. Consistent with this, the present study demonstrated that centers with a certification duration of more than two years—and thus repeated annual recertifications—were associated with more favorable patient outcomes than recently certified centers (HR 0.98 vs. HR 0.90). An extrapolation of the absolute risk reduction to all newly diagnosed head and neck cancer patients in Germany in 2017 who were not treated in certified centers would result in a potential of 2,343 life years that was not exploited [26], the difference in survival for SHI data results in an absolute risk reduction of more than 1.8 months per patient. Based on the available results and previous publications relating to other entities, it can be assumed that the continued long-term certification of head and neck tumor centers (> 5 years) will lead to an even greater difference. On the other hand, it is possible that hospitals that sought certification at an early stage had particularly good prerequisites and thus the effect on patient outcomes may be particularly high.

The benefits of the certification programme were recognized at an early stage. By 31.12.2023, 79 head and neck tumor centers at 81 locations (including seven centers abroad: four centers in Switzerland and 3 in Austria) had been certified. According to the annual report of the German Cancer Society, 56.8% of the 11,577 primary cases in 2022 were treated in certified centers [2] in comparison to 55.27% in 2023. Although the number of centers doubled, the number of patients treated in certified centers increased by only just under 20% over a five-year period (2017–2022). There has been no further upward trend since 2022. Due to the significant increase in the number of centers and the aforementioned effect of the progression of survival benefits after more than five years, a renewed scientific examination or monitoring of these center effects beyond the scope of the WiZen study would be desirable. The available data showed a non-significant trend indicating that early and long-standing certification appear to have a positive association with treatment in head and neck tumor centers. One possible reason for the lack of significance is the relatively small number of centers.

The assumption that longer continuity of certification improves the survival outcome is supported by the analyzed effects of certified centers treating other entities [19, 27–29].

The present study demonstrated further advantages in addition to improved survival time. When subgroups are formed according to the UICC stage, patients with UICC stages I–III have significantly better overall survival (HR 0.79) and recurrence-free survival after R0 resection (HR 0.81) if they are treated at certified centers (*p <* 0.001 s. Table 2). Although an international comparison is significantly more challenging due to variations in healthcare systems, certification programs, and potential differences in national guidelines, the outcomes align with data from Japan [30]. In this study, the 3-year survival rate following treatment at authorized (86.6%) and non-authorized (78.8%) hospitals was investigated. These associations were clearly evident for UICC stages I–III. However, the evaluation of treatment based on survival in UICC stage IV should certainly be viewed critically. In a palliative setting, survival should not be the only criterion for evaluating treatment quality.

Functional outcome and quality of life aspects are essential especially for head and neck cancer patients. Functions such as speaking, swallowing, smelling, and breathing – often taken for granted—can become profoundly challenging for patients undergoing treatment for head and neck cancer. The aim to have a complete resection with save margins can often jeopardize these functional aspects thus an interdisciplinary discussion about the best approach might stand in contrast to perform surgical resection as soon as possible after diagnosis. The analysis of SHI data revealed statistically significant differences in the use of resection as an index treatment between certified and non-certified centers. Initial treatment with resections was more commonly documented in non-certified institutions, especially in patients under 80 years of age, men, individuals with pharyngeal carcinomas, and those treated in high-volume hospitals. These differences suggest variations in clinical decision-making that may reflect differences in guideline adherence, availability of multimodal treatment options, or the absence of structured interdisciplinary processes. However, this should not be misinterpreted as undervaluing surgery; rather, it emphasizes the importance of structured, multidisciplinary and evidence-based decision-making. This allows patients to be selected for surgical therapy with a high chance of success.

Current evidence underscores the role of certification and multidisciplinary tumor boards in improving the quality of oncologic care. Certified centers are associated with better adherence to evidence-based guidelines, more frequent interdisciplinary case discussions, and improved outcomes in head and neck cancer patients [31]. For example, guideline-based recommendations from the German S3 guideline emphasize individualized, multimodal treatment planning through tumor boards, particularly in anatomically and functionally complex regions such as the pharynx [7–9].

The observed preference for primary surgical approaches in non-certified centers may reflect limited access to radiotherapy or chemoradiation facilities, or institutional cultures favoring surgical intervention. Similar trends have been reported in studies showing that structural characteristics of hospitals — such as certification status, case volume, and presence of comprehensive cancer centers — can significantly influence treatment decisions and outcomes [32].

Furthermore, an analysis of the number of beds revealed that large clinics are more likely to be certified. This can be attributed to the ever-increasing requirements resulting from the refinement and numerical adjustment of quality indicators (e.g. necessary collaborations, proportion of study patients, etc.), as well as cost pressures and the associated personnel costs. Large hospitals are better placed to offset additional costs if the DRG system does not adequately cover them [33].

From a health policy perspective, these findings highlight the importance of promoting certification processes and ensuring equitable access to interdisciplinary care structures across all hospital types. Efforts to expand the certification system and integrate smaller or non-certified institutions into regional cancer networks could reduce treatment variability and improve care quality. Activities of this kind have been, and continue to be, successfully promoted in Germany and at the European level (compare to European cancer centers [34–36]). This enables the potential benefits of certification for patients to be utilized also in the wider context.

Future research should evaluate the clinical outcomes associated with these observed treatment patterns and further investigate the institutional and systemic factors driving them. Linking administrative data with clinical registries may provide deeper insights into the quality and effectiveness of care across different hospital settings. In addition, evaluation of the data reveals a more comprehensive picture of the cohorts in certified centers, with the same structure of patient characteristics. This enables transparent reporting of treatment outcomes and may enhance national and international comparability.

### Limitation and strengths

The analyzed cohort exhibited a low degree of selectivity, as the documentation of SHI billing data was subject to statutory provisions, while the CRC data was subject to mandatory reporting under the Cancer Screening and Registry Act (KFRG). The CCR data was already highly complete before the KFRG. Additionally, a large number of patient-specific confounding factors, such as comorbidities or disease severity information, could be included for both data sources. For SHI data, characteristics of the treating hospitals were also considered. Unfortunately, a number of the influencing variables were only available for one data source, and information on socioeconomic status was not available.

Since volume can impact relevant outcomes such as survival [37–39], and since a minimum volume is required for DKG certification, some of the present results could be due to volume effects. However, since the SHI data came from a single health insurance company and the main treatment clinic allocation was not available for all patients in the CCR data, volume data were unavailable and it was not possible to quantify the total patient volume in the respective treatment clinics and include it in the analysis. Although an adjustment was made based on the number of beds in the hospital, this information could only partially compensate for the lack of volume data. Consequently, the potential for bias due to unmeasured confounding could not be completely eliminated. The same applies to other possible influential factors that are not included in the data, such as socioeconomic status, detailed hospital resources and case mix.

Assigning the certification status of individual clinics to their entire network carries the risk of misclassification and can also result in an overly conservative estimate of the potential certification effect. This may also be due to the fact that follow-up treatment in certified centers for patients who received initial treatment in non-certified clinics has not yet been thoroughly mapped, which requires further analysis. Furthermore, hospitals typically restructure their processes and systems during the certification process. To avoid misclassification of treatments, patients whose treatment was carried out one year prior to the issuance of the certificate were excluded.

The status of 'certification' as a complex structure of interventions at the level of the treating institutions can be difficult to quantify. On the other hand, randomization of the cohort was not possible due to the structure of the certification system and the use of retrospective secondary/cancer registry data. Thus, it is not possible to draw definitive conclusions in terms of causal inference. Nevertheless, by using different data sources and comprehensively considering relevant patient, tumor and hospital characteristics in the sense of risk adjustment, the association of certification status with patient survival could be investigated validly. This minimized the risk of bias and enabled the effect of certification to be compared across different types of cancer. Additionally, several of Bradford Hill's criteria for causality [40], such as consistency of association, temporal sequence, dose–response relationship and coherence, can be considered to have been met. This indicates that the statements and conclusions may be considered to have a high degree of reliability.

## Conclusion

The present analysis of the comprehensive WiZen cohort data on head and neck malignancies demonstrated a robust survival advantage for patients treated at certified head and neck tumor centers compared with those treated at a non-certified hospital. These data underline the validity of the certification programme and the associated improvement in the treatment of head and neck tumor patients at certified centers, particularly for tumor stages UICC I—III. The exact reasons for the improvement in treatment appear to be complex and require further investigation.

## Supplementary Information

Supplementary Material 1.

## Data Availability

The authors confirm that the data utilized in this study cannot be made available in the manuscript, the supplemental files, or in a public repository due to German data protection laws (‘Bundesdatenschutzgesetz’, BDSG). Therefore, they are stored on a secure drive in the WIdO, to facilitate replication of the results. Generally, access to data of statutory health insurance funds for research purposes is possible only under the conditions defined in German Social Law (SGB V § 287). Requests for data access can be sent as a formal proposal specifying the recipient and purpose of the data transfer to the appropriate data protection agency. Access to the data used in this study can only be provided to external parties under the conditions of the cooperation contract of this research project and after written approval by the sickness fund. For assistance in obtaining access to the data, please contact wido@wido.bv.aok.de.

## Abbreviations

ADT: Arbeitsgemeinschaft Deutscher Tumorzentren (Association of German Tumor Centers)
AOK: Allgemeine Ortskrankenkasse (General Local Health Insurance Fund)
BDSG: Bundesdatenschutzgesetz (German data protection law)
BMBF: Bundesministerium für Bildung und Forschung (Federal Ministry of Education and Research)
BMG: Bundesministerium für Gesundheit (Federal Ministry of Health)
CCR: Clinical cancer registry
CI: Confidence interval
CUP: Carcinoma of unknown primary
DKG: Deutsche Krebsgesellschaft (German Cancer Society)
DKH: Deutsche Krebshilfe (German Cancer Aid)
DRG: Diagnosis Related Groups
EU: European Union
G-BA: Gemeinsamer Bundesausschuss (Joint Federal Committee)
HR: Hazard ratio
ICD: International statistical classification of diseases and related health problems
IQTIG: Institut für Qualitätssicherung und Transparenz im Gesundheitswesen (Institute for Quality Assurance and Transparency in Health Care)
KFRG: Krebsfrüherkennungs- und -registergesetz (cancer screening and registry act)
SGB: Sozialgesetzbuch (social code book)
SHI: Statutory health insurance
TZR: Tumorzentrum Regensburg am Zentrum für Qualitätssicherung und Versorgungsforschung an der Universität Regensburg (Regensburg Tumor Center at the Center for Quality Assurance and Health Services Research at the University of Regensburg)
UICC: Union internationale contre le cancer
WIdO: Wissenschaftliches Institut der AOK (AOK Research Institute)
WiZen: Wirksamkeit der Versorgung in onkologischen Zentren (Effectiveness of care in certified cancer centers)
ZEGV: Zentrum für Evidenzbasierte Gesundheitsversorgung (Center for Evidence-based Healthcare)
