# Supplementary material for: The effects of certification of head and neck cancer centers on the survival of patients with a head and neck cancer
Source: BMC Cancer. 2026 Jan 26;26:132. doi: 10.1186/s12885-026-15624-z (PMC12837409; doi:10.1186/s12885-026-15624-z)
Supplement: Supplementary file 1 — Supplementary Material 1. [file 12885_2026_15624_MOESM1_ESM.docx]

**Additional file 1: Supplementary material**

**The effects of certification of head and neck cancer centers on the survival of patients with a head and neck cancer**

**Olaf Schoffer^1^, Max Kemper^2^, Michael Gerken^3^, Veronika Bierbaum^1^, Christoph Bobeth^1^, Martin Rößler^1^, Patrik Dröge^4^, Thomas Ruhnke^4^, Christian Günster^4^, Kees Kleihues-van Tol^5^, Chia-Jung Busch^6^, Monika Klinkhammer-Schalke^3,5^, Jochen Schmitt^1^**

*1 Center for Evidence-Based Healthcare, Faculty of Medicine and University Hospital Carl Gustav Carus, TUD Dresden University of Technology, Fetscherstraße 74, 01307 Dresden, Germany
2 Department of Otorhinolaryngology, Head and Neck Surgery, Faculty of Medicine Carl Gustav Carus, University Hospital Dresden*, *TUD Dresden University of Technology, Fetscherstraße 74, 01307 Dresden, Germany
3 Tumor center - Center for Quality Assurance and Health Services Research, University of Regensburg, Am BioPark 9, 93053 Regensburg, Germany*
*4 AOK Research Institute (WIdO), Rosenthaler Str. 31, 10178 Berlin, Germany*
*5 Association of German Tumor Centers (ADT), Kuno-Fischer-Straße 8, 14057 Berlin, Germany
6 Department of Otorhinolaryngology, Head and Neck Surgery, University Medicine Greifswald, Ferdinand-Sauerbruch-Strasse, 17475 Greifswald, Germany*

**Supplement Table 1**: Sensitivity analysis – hazard ratio of the adjusted certification effect for subgroups based on the data (SHI: Statutory Health Insurance; CCR: Clinical Cancer Registry)

| data source | subgroups | | HR | 95%-CI |
| --- | --- | --- | --- | --- |
| SHI^1^ | **gender** | **female** | 0.96 | (0.86-1.06) |
|  |  | **male** | 0.93* | (0.87-0.99) |
|  | **secondary cancer** | **yes** | 0.94 | (0.86-1.02) |
|  |  | **no** | 0.94 | (0.87-1.01) |
|  | **distant metastases** | **yes** | 0.91 | (0.80-1.04) |
|  |  | **no** | 0.95 | (0.90-1.01) |
|  | **tumor resection** | **yes** | 0.95 | (0.88-1.02) |
|  |  | **no** | 0.92 | (0.84-1.01) |
|  | **single hospital** | **yes** | 0.97 | (0.92-1.03) |
|  |  | **no** | Not estimable | |
|  | **number of hospital beds** | **<500** | 0.95 | (0.89-1.00) |
|  |  | >=**500** | 0.87 | (0.68-1.12) |
|  | **localisation** | **oral cavity, salivary glands C00-08** | 0.93 | (0.86-1.01) |
|  |  | **pharynx C09-14** | 0.98 | (0.89-1.07) |
|  |  | **nose, sinuses, ear, larynx C30-32** | 0.94 | (0.84-1.06) |
| CCR^2^ | **gender** | **female** | 0.97 | (0.84-1.12) |
|  |  | **male** | 0.87*** | (0.81-0.94) |
|  | **age at diagnosis** | **0- 49** | 0.81 | (0.64-1.01) |
|  |  | **50-59** | 0.86* | (0.76-0.97) |
|  |  | **60-69** | 0.95 | (0.82-1.04) |
|  |  | **70-79** | 0.87* | (0.75-0.99) |
|  |  | **80+** | 1.06 | (0.87-1.29) |
|  | **localisation** | **oral cavity, salivary glands C00-08** | 0.86** | (0.79-0.95) |
|  |  | **pharynx C09-14** | 0.90* | (0.81-0.99) |
|  |  | **nose, sinuses, ear, larynx C30-32** | 0.94 | (0.80-1.09) |

HR=Hazard ratio, CI=95% confidence interval, p-value: *p<5%, **p<1%, ***p<0.1%

1: adjusted for age, gender, distant metastasis, other oncological disease, Elixhauser comorbidities, number of hospital beds, teaching hospital, university hospital, hospital sponsorship, year of index treatment - dummy-coded (basis: SHI data)

2: adjusted for gender, age of diagnosis, year of diagnosis, ICD-10 diagnosis, stage, grading, lymph vessel invasion, venous invasion (basis: SHI data)

**Supplement table 2**: Sensitivity analysis - hazard ratio of the adjusted certification effect according to the duration of certification (basis: statutory health insurance data)

| duration of certification | HR^1^ | 95%-CI |
| --- | --- | --- |
| (ref: not certified) | - | - |
| <1 year | 0.98 | (0.91-1.05) |
| 1-<2 years | 0.94 | (0.86-1.02) |
| 2-<5 years | 0.90* | (0.83-0.98) |
| 5 or more years | 0.82 | (0.61-1.09) |

^1^: adjusted for age, gender, distant metastasis, other oncological disease, Elixhauser comorbidities, number of hospital beds, teaching hospital, university hospital, hospital ownership, year of index treatment - dummy-coded
